# The use of cost-effectiveness analysis for health benefit package design – should countries follow a sectoral, incremental or hybrid approach?

**DOI:** 10.1186/s12962-023-00484-2

**Published:** 2023-10-09

**Authors:** Rob Baltussen, Gavin Surgey, Anna Vassall, Ole F. Norheim, Kalipso Chalkidou, Sameen Siddiqi, Mojtaba Nouhi, Sitaporn Youngkong, Maarten Jansen, Leon Bijlmakers, Wija Oortwijn

**Affiliations:** 1 Radboud University Medical Center, P.O. Box 9101, Nijmegen, 6500 HB The Netherlands; 2 London School of Hygiene and Tropical Medicine, London, UK; 3 University of Bergen, Bergen, Norway; 4 The Global Fund, Geneva, Switzerland; 5 Imperial College, London, UK; 6 Aga Khan University, Karachi, Pakistan; 7 Ministry of Health and Medical Education, Tehran, Iran; 8 Tehran University of Medical Sciences, Tehran, Iran; 9 Mahidol University, Bangkok, Thailand

**Keywords:** Cost-effectiveness analysis, Health benefit packages, Universal health coverage, Incremental analysis, Sectoral analysis

## Abstract

**Background:**

Countries around the world are increasingly rethinking the design of their health benefit package to achieve universal health coverage. Countries can periodically revise their packages on the basis of sectoral cost-effectiveness analyses, i.e. by evaluating a broad set of services against a ‘doing nothing’ scenario using a budget constraint. Alternatively, they can use incremental cost-effectiveness analyses, i.e. to evaluate specific services against current practice using a threshold. In addition, countries may employ hybrid approaches which combines elements of sectoral and incremental cost-effectiveness analysis - a country may e.g. not evaluate the comprehensive set of all services but rather relatively small sets of services targeting a certain condition. However, there is little practical guidance for countries as to which kind of approach they should follow.

**Methods:**

The present study was based on expert consultation. We refined the typology of approaches of cost-effectiveness analysis for benefit package design, identified factors that should be considered in the choice of approach, and developed recommendations. We reached consensus among experts over the course of several review rounds.

**Results:**

Sectoral cost-effectiveness analysis is especially suited in contexts with large allocative inefficiencies in current service provision and can, in theory, realize large efficiency gains. However, it may be challenging to implement a comprehensive redesign of the package in practice. Incremental cost-effectiveness analysis is especially relevant in contexts where specific new services may impact the sustainability of the health system. It may potentially support efficiency improvement, but its focus has typically been on new services while existing inefficiencies remain unchallenged. The use of hybrid approach may be a way forward to address the strengths and weaknesses of sectoral and incremental analysis areas. Such analysis may be especially useful to target disease areas with suspected high inefficiencies in service provision, and would then make good use of the available research capacity and be politically rewarding. However, disease-specific analyses bear the risk of not addressing resource allocation inefficiencies across disease areas.

**Conclusions:**

Countries should carefully select their approach of cost-effectiveness analyses for benefit package design, based on their decision-making context.

## Background

Many countries are rethinking the design of their health benefit packages as a means to support the progressive realisation of universal health coverage (UHC), i.e. to select the appropriate set of services at fair levels of coverage and financial protection [1–3].

In 2000, the World Health Organisation (WHO) issued conceptual guidance on health benefit package design and distinguished de facto two broad approaches – both centered around the use of cost-effectiveness analysis [4]. First, countries can review their health benefit package periodically on the basis of *sectoral cost-effectiveness analyses* i.e. by evaluating a broad set of services against a ‘doing nothing’ scenario using a budget constraint. Various countries, including Ethiopia and Pakistan recently, [3, 5] embarked on such an approach, supported by the Disease Control Priorities (DCP) project and informed by global databases such as the DCP registry, the Global Health Cost-Effectiveness Analysis Registry (GHCEAR), and WHO-CHOICE. Such analyses typically lead to the definition of a health benefit package including a large number of services that are either publicly funded or as part of an insurance package [3, 5]. Second, countries can rely on *incremental cost-effectiveness analyses* to inform the design of their benefit packages, i.e., by evaluating specific services against ‘current practice’ using a cost-effectiveness threshold. Taking an incremental analysis approach to benefit package design is implied by many guidelines, [6–8] organizations (e.g. international Decision Support Initiative) and networks (e.g. European Network for Health Technology Assessment) that support countries in the evaluation of health services. Such analyses typically lead to a decision to in- or exclude single services to a health benefit package [6–8]. In addition to these formally defined approaches, countries employ a wide array of alternative hybrid approaches incorporating elements of both sectoral and incremental analysis e.g. to improve the allocative efficiency of disease focussed programmes [9].

Now, 20 years later, there is still little practical guidance for countries as to which kind of approach they should follow. This is remarkable as the optimal choice depends on a variety of factors such as the nature of allocative inefficiencies, quality of analysis, political feasibility of reallocation decisions and integrated health system analysis. This paper fills this gap and defines sectoral and incremental cost-effectiveness analysis for designing a benefit package by characterising their scope, comparator and the way opportunity costs are considered (Sect. 2) and reports on factors to consider when choosing between these approaches (Sect. 3). For the purpose of comparison and contrast, the paper deliberately takes a somewhat extreme perspective in its presentation of sectoral and incremental approach as the two principal approaches for benefit package design. We realize countries often use hybrid approaches and we also present their merits (Sect. 4). Finally, we conclude with several recommendations (Sect. 5).

The paper does not claim to be comprehensive but is rather meant as a structure overview that draws on literature and expert consultation. Its aim is to create a better understanding of the different approaches and to support countries towards making an informed choice. Much of the argumentation in this paper is centred around the use of cost-effectiveness analysis because of its important role in health benefit package design. Yet, the paper is also relevant to the broader perspective of progressive realization of UHC, in which also other considerations such as population coverage, equity and financial protection are taken into account [10].

## Methods

The present study was based on expert consultation. The process, spanning a period of two years, started with an invitation by two lead authors to several leading experts on health benefit package design from academic institutes in low-, middle- and high-income countries to participate in the review on the use of sectoral versus incremental cost-effectiveness analysis. These experts are included as co-authors of the paper. The lead authors developed a first typology of approaches for cost-effectiveness analysis for benefit package design based on the paper by the WHO in 2000 [4]. A series of subsequent discussion / review rounds took place through emails, conference calls and bilateral interactions, in which all authors (i) commented on this typology; (ii) were asked to identify literature and then confirm or redefine the different approaches; (iii) identified factors that should be considered in choosing a suitable approach; (iv) identified country case studies to illustrate the argumentation, and (v) developed recommendations. In each round, the lead authors summarized the comments, proposed revisions to the initial manuscript, which were then approved, rejected or commented on by co-authors. We reached consensus on the typology of approaches and factors to consider over the course of several review rounds. No experts other than the study authors were involved in the process. The views expressed in this paper are not meant to be representative of all researchers in the area of benefit package design, and the resulting methodological guidance should also be interpreted as such.

## Results

### Sectoral and incremental analysis

**Fig. 1:**
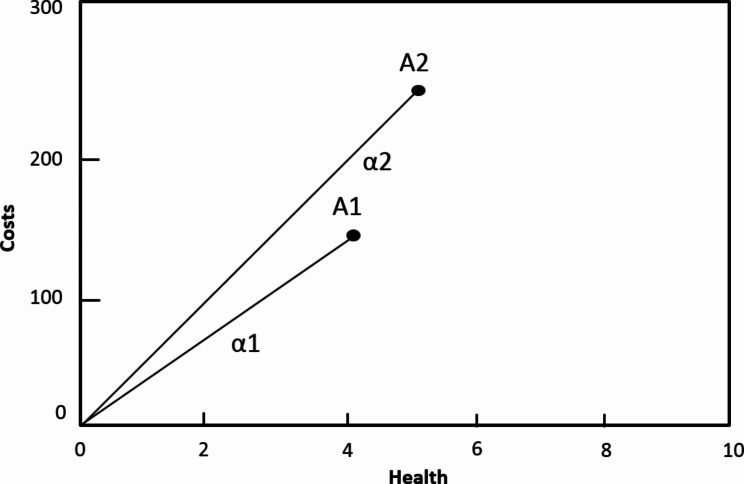
Average cost-effectiveness ratios in sectoral analysis^*^. (* The origin ‘0’ reflects the ‘doing nothing’ comparator. The slopes α1 and α2 reflect the average cost-effectiveness ratios respectively of the existing service A1 and a new service A2)

For the purpose of this paper, we interpret sectoral cost-effectiveness analysis in its most explicit form, i.e. on the basis of three key characteristics (Table 1). Firstly, in terms of scope, sectoral analysis evaluates *in an integrated effort a comprehensive set of services to support decisions in the (re)design of the entire health benefit package*. Hence, sectoral analysis can be considered as a joint set of decisions on services, which together establish the revised benefit package. Secondly, in terms of comparator, sectoral analysis evaluates costs and effects of a set of *new and existing services in comparison to ‘doing nothing’* (also known as Generalised cost-effectiveness analysis). The ‘doing nothing’ comparator reflects a hypothetical situation in which the set of services currently being provided is stopped, thereby creating an analytical scenario in which all services could potentially be reallocated and considered for inclusion. Costs and effects of independent services are commonly reported in average cost-effectiveness ratios. This is illustrated in Fig. 1, where A1 reflects current practice. The slopes α1 and α2 reflect the average cost-effectiveness ratios respectively of the existing service A1 and a new service A2. In terms of opportunity costs, analysts can rank order services based on these ratios, following decision rules as described elsewhere [4]. The most efficient services are accordingly included in the benefit package until the budget is exhausted. This will maximize health gains for a given budget and likely result in transferring resources from less efficient to more efficient services. Thirdly, this implies that sectoral analysis employs an *explicit budget constraint to* reflect opportunity costs of included services and expresses these in terms of foregone health gains of the excluded services. Budget constraint here refers to the total sum of financial resources available to fund the benefit package.

**Table 1:** Summary of characteristics of different approaches to benefit package design

Approach		Characteristics										
		Scope		Comparator program		Reflection of opportunity costs						
Sectoral analysis		Entire benefit package		‘Doing nothing’		Budget constraint						
Incremental analysis		Specific service		‘Current practice’		Cost-effectiveness threshold						
Hybrid approach		Sets of specific services		Flexible		Flexible						

**Fig. 2:**
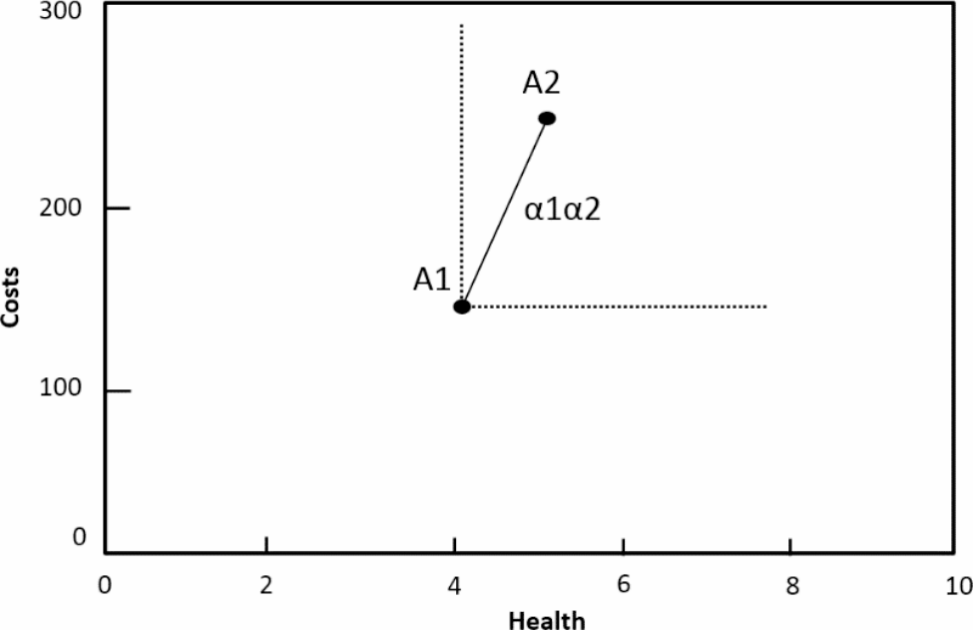
Incremental cost-effectiveness ratios in incremental analysis^*^. (* Incremental analysis typically compares a new intervention A2 to standard care A1, and the incremental cost-effectiveness ratio is reflected by the slope α1α2. Incremental analysis may also evaluate standard care but this is not often done. In the figure, it would entail the comparison of A1 to the ‘doing nothing’ scenario. In that case, the incremental cost-effectiveness is reflected by the slope from the origin to A1)

We also define incremental cost-effectiveness analysis by its key characteristics (Table 1). Firstly, in terms of scope, incremental analysis supports *decision making at the margin*, i.e., adding or removing a specific service to the current service mix. The use of incremental analysis for health benefit package design can thus best be interpreted as a series of separate decisions on specific services, which together over time revise the benefit package. As an example, Thailand routinely uses incremental analysis to define its health benefit package and annually undertakes some 20 analyses [11, 12]. Secondly, in terms of comparator, incremental analysis evaluates the costs and effects of a specific service *against the current service mix (*i.e. standard care) [13]. The results are expressed in an incremental cost-effectiveness ratio. This is illustrated in Fig. 2 where A1 reflects current practice, and the slope α1α2 reflects the incremental cost-effectiveness ratios of the service A2. Thirdly, in terms of opportunity costs, incremental analysis compares the incremental cost-effectiveness ratios to a *cost-effectiveness threshold*. In the example, the incremental cost-effectiveness ratio α1α2 is compared to this threshold to assess the opportunity costs of including A2 in the health benefit package. In case it compares favourably, the inclusion of a service is considered to increase the overall efficiency of the health benefit package. There are different approaches to estimate a country’s cost-effectiveness threshold [14, 15].

### Factors to consider when choosing between sectoral and incremental cost-effectiveness analysis

#### Nature of allocative inefficiencies

Sectoral analysis is especially geared towards the evaluation of existing services and is therefore especially suited in contexts with *large allocative inefficiencies in current service provision*. Such inefficiencies may relate to the inefficiency of the service itself (e.g. the treatment of advanced stages of chronic obstructive pulmonary disease in the African region), [16] by whom the service is provided (e.g. by highly trained healthcare professionals or by health workers with fewer qualifications) or where the service is provided (e.g. the hospital or in the community). Several international databases such as the DCP registry, GHCEAR and WHO-CHOICE support the use of sectoral analysis and invariably report large variations in cost-effectiveness ratios of services. This indicates the large potential for allocative efficiency gains in a country when existing services are replaced by new, more efficient, services. Moreover, sectoral analysis evaluates a comprehensive set of services in a single concentrated effort and can therefore be hypothesized to lead to relatively high efficiency gains in a given time period. However, it may be challenging to implement such a comprehensive redesign of the package, [2] and we do not know of evidence to demonstrate that sectoral analysis has indeed realized large efficiency gains in practice.

As noted, incremental analysis supports decision making at the margin as to whether a new service should replace an existing one, or an existing service should be removed. However, in practice, the use of incremental analysis is heavily skewed towards the evaluation of new services [17]. In this use, incremental analysis is especially relevant in contexts where *specific new services raise challenges to the allocative efficiency* and sustainability of the health system. Incremental analysis is then used to prevent the adoption of inefficient new services or to support price negotiations on these. An example is the evaluation of dialysis in end-stage renal disease for coverage decisions in Thailand [18]. There is evidence, although variable, that incremental analysis as part of health technology assessment (HTA) studies had impact on coverage decisions and may have led to considerable cost savings [19]. However, incremental analysis rarely evaluates whether the existing services themselves are worth doing - and thereby takes as a starting point that some services addressing a certain condition will always be undertaken. The rare use of incremental analysis to evaluate existing services is not a weakness of the method itself but rather of its application in practice [20]. Yet, it means that, in the present use of incremental analysis, large allocative inefficiencies may remain unchallenged.

#### Quality of analysis

The analytical demand for sectoral analysis is much larger than that for incremental analysis and this may challenge the quality of analysis. Sectoral analysis may entail the evaluation of up to say 200 services whereas incremental analysis typically evaluates a single service. Moreover, analyses need to take into account that the cost-effectiveness of a service is often dependent on the availability of another service. For example, the cost and effects of malaria treatment are dependent on whether malaria bed nets are in place [21] [21]. In sectoral analysis these interactions between services are numerous and require detailed study which is difficult to account for in large scale sectoral analysis. In addition, the large scale of sectoral analysis may compromise the time and resources available for a careful judgment of the value of services in consultation with stakeholders, and therefore the quality of analysis. For example, whereas an appraisal committee in the context of incremental analysis (such as in the Netherlands) [22] may spend half a day to deliberate on a specific service, this timeframe may be much shorter in the case of sectoral analysis when over the course of several days a large set of services needs to be appraised.

Both sectoral and incremental analyses often draw on international databases on cost-effectiveness, such as the DCP registry, GHCEAR and WHO-CHOICE. Yet, there are challenges to applying estimates from these databases to local situations. Firstly, the definition of services included in these databases may be very different from those implemented at the country level, in terms of e.g. platform and treatment regimen. Secondly, the databases include a complex mix of studies, employing different comparators. The WHO-CHOICE database evaluates services in comparison to ‘doing nothing’ and expresses these in average cost-effectiveness ratios. In contrast, both the DCP registry and GHCEAR are based on literature reviews of international studies: most of these studies compare services to current practice and render incremental rather than average cost-effectiveness ratios. A case in point here is GHCEAR which often classifies comparators in studies as ‘doing nothing’ which is understood as doing nothing compared to current practices instead of the null scenario as WHO-CHOICE does. For example, the evaluation of Rotavirus vaccination employs ‘no vaccination’ as comparator, which is categorized as ‘doing nothing’, but in reality, patients receive ‘current practice’ treatment [17]. Analysts should be aware of these differences and be careful to correctly interpret the ratios produced by WHO-CHOICE, DCP, and GHCEAR. This is illustrated in Figs. 1 and 2 by the difference in incremental (α1-α2) and average (α2) cost-effectiveness ratios for service A2.

Thirdly, incremental analysis is highly context-specific and is difficult to transfer across countries. Whereas differences in disease burden, health service use, and price levels of resource inputs can somehow be adjusted, [23] it is much more challenging to adjust for the comparator program ‘current practice’. For this reason, analysts should be cautious of applying international estimates on incremental cost-effectiveness of services to their own setting. In contrast, sectoral analysis compares services to ‘doing nothing’ which is assumed similar across countries - this means that a country can use international evidence on average cost-effectiveness ratios after adjusting for contextual factors such as differences in epidemiology, prices etc [4]. Fourthly, an extra complication to the use of incremental cost-effectiveness analysis is that it relies on cost-effectiveness thresholds which are hard to estimate and surrounded by large uncertainties [14].

#### Political feasibility of reallocation decisions

Resource reallocation decisions are politically notoriously difficult to make because of e.g. powerful interest groups, politicians or bureaucrats who pursue their own objectives, voting pressures and institutional arrangements [24, 25]. While this is true for both sectoral and incremental analyses in a general sense, there are certain aspects in both analysis that may impact the political feasibility of reallocation decisions.

Incremental analyses commonly employ a cost-effectiveness threshold to inform decisions on the in- or exclusion of specific services. Theoretically, this threshold serves to reflect the opportunity costs of the decision, i.e. the health foregone or gained because resources are transferred from one service to another. However, in practice, opportunity costs are rarely specified and therefore remain largely intangible [26]. The lack of tangibility of any losses may influence how the public interprets benefit package decisions, and how politicians may react. The resulting effect may well be that the inclusion of new services is disproportionally favoured as it is not clear which services are displaced. Likewise, the exclusion of existing services is disproportionally discouraged as it is not clear which services are implemented in return.

The use of sectoral analysis may invoke different political dynamics. First, because decisions are taken on many services simultaneously, it involves many winning and losing stakeholders, and thereby potentially larger public debate and resistance in comparison to incremental analysis. Second, as decisions to remove services from the benefit package are accompanied by explicit decisions to add other services sectoral reallocation may face less general public resistance- because it is clear which health gains are realised as a result of reallocation. In a similar vein, investment in new inefficient services are always implying the displacement of existing services. It can be hypothesised that this provides an important counterbalance to public pressure to include emerging new services in the package.

#### Integrated health system analysis

Effective service implementation is largely dependent on a well-functioning health system, but it is analytically challenging to capture interactions between services and the health system [27]. Sectoral analysis can in principle improve on this: (i) it allows an evaluation of the feasibility of a planned health benefit package by comparing the total required human and material resources with the available capacity – and by prioritizing services on that basis if necessary; (ii) sectoral analysis can compare costs and effects of investments in services with investments in health system strengthening; and in doing so it can make the need for health system investments more explicit. However, incorporating these issues in sectoral analysis is challenging, and no country has yet realized this.

Incremental analysis typically takes the prevailing health system as a given, although efforts are being made to consider health system constraints that may obstruct service delivery (e.g. shortage or skills of certain health workers) in HIV control [28]. Yet, these are not yet routinely used [29].

### Hybrid analyses

In practice, many countries employ a hybrid approach in which analysts adopt different varieties in terms of the scope and comparator of analysis, and how they deal with opportunity costs. In terms of scope, one option is that a country does not evaluate the comprehensive set of all services but rather relatively small sets of services targeting a certain condition. This approach was used in the economic analysis of hypertension control in Ghana which involved several treatment regimens [30]. It is being applied in the Netherlands to routinely review existing and new services in consecutive disease areas (and which was estimated to lead to large savings for knee and hip arthrosis) [31]. A further focus in this option is to only evaluate services with large social impact (e.g. in terms of budget). This approach is used to define the health benefit package in Ukraine addressing four priority health conditions, [32] in Iran with regards to six major disease areas, [33] and in Thailand for population-based screening [34].

How well does such hybrid analysis perform with respect to the mentioned factors? In terms of *nature of allocative inefficiencies*, hybrid analysis may be especially useful to target disease areas with suspected high inefficiencies (in either current or new services). However, it should be realised that disease-specific analyses bear the risk of not addressing issues of allocation between disease areas. In terms of *quality of analysis*, hybrid analysis makes good use of the available research capacity in a country by its targeted approach. The use of hybrid analysis may positively affect the *political feasibility of reallocation decisions* as the focus on a certain disease domain may be politically rewarding, may help to reach certain national goals, or may trigger less opposition among patients as it concerns replacement of services within the clinical pathway of a condition. Finally, hybrid analysis can potentially capture interactions between services and the *health system*, obviously depending on the scope of analysis.

Another form of hybrid analysis is the sequential combination of sectoral and incremental analysis. That is, countries can review their entire benefit package periodically by performing sectoral analyses, followed by incremental analyses to decide on the in- or exclusion of specific services when the occasion arises.

## Discussion

This paper compares the use of sectoral, incremental and hybrid analysis to support countries in the design of their health benefit package. Countries should carefully balance the advantages and disadvantages of each approach and consider the relevant fit within their decision-making context.

Sectoral analysis is especially suited in contexts with large allocative inefficiencies in current service provision and can, in theory, realize large efficiency gains. However, it may be challenging to implement a comprehensive redesign of the package in practice. Incremental analysis is especially relevant in contexts where specific new services raise challenges to the allocative efficiency and sustainability of the health system. It may potentially support efficiency improvement but its focus has typically been on new services while existing inefficiencies remain unchallenged. A key challenge for both incremental and especially sectoral analysis is the quality of the available evidence base. The use of hybrid approach may be a way forward to address the strengths and weaknesses of both sectoral and incremental analysis.

This paper has shown that the availability of evidence is a central challenge in sectoral, incremental and hybrid analyses. We propose an alternative use of international evidence on cost-effectiveness. We argue that if studies consistently demonstrate - across a wide variety of settings possibly using a variety of ‘current practices’ as comparator programmes - that a certain service is cost-effective, there is a likelihood that it will be cost-effective in any decision-making context (both in terms of incremental and average cost-effectiveness). For example, studies in several countries have repeatedly confirmed the cost-effectiveness of the treatment of drug susceptible tuberculosis [35]. We recognize that this approach does not provide detailed cost-effectiveness estimates but only broad indications on cost-effectiveness – this corresponds with our view that cost-effectiveness analysis should not be used formulaically [36] and that broad classifications of cost-effectiveness are sufficient input for health benefit package design.

An important observation in this paper is the political dynamics of benefit package design, related to the use of a cost-effectiveness threshold in incremental analysis and a budget constraint in sectoral analysis. The use of a budget constraint makes opportunity costs explicit, and we recommend countries, as much as possible, to employ this in their analysis. However, we are aware that such budget constraints / fiscal space are not always known and well-defined.

While this paper relies heavily on the guidance issued by the WHO in 2000 [4], the literature on the use of cost-effectiveness analysis for broad sectoral reallocation decisions traces back to the 1970’s when Milton, Weinstein, and Stason published their seminal paper ‘The foundations of cost-effectiveness analysis for Health and Medical Practices’ [37]. The work by WHO and our paper, alongside numerous other publications on the use of cost-effectiveness analysis, can be considered as ongoing efforts to put the observations from Milton et al. in practice.

Finally, much of the argumentation of this paper is based on cost-effectiveness analysis. Yet, we recommend countries to also take into account other fairness considerations to adequately achieve progressive realization of UHC [10] and as such refer to procedural guidance for doing so [38].

## Data Availability

Not applicable.
